# Supplementary material for: CORGIAS: identifying correlated gene pairs by considering evolutionary history in a large-scale prokaryotic genome dataset
Source: NAR Genom Bioinform. 2025 Dec 12;7(4):lqaf182. doi: 10.1093/nargab/lqaf182 (PMC12699329; doi:10.1093/nargab/lqaf182)
Supplement: lqaf182_Supplemental_Files [file lqaf182_supplemental_files.zip › TableS1-S7.pdf]

Supplementary Table S1. PRAUC of ASA and SEV using various methods for ancestral state reconstruction. "MPPA" is the maximum likelihood method, and the other are the maximum parsimony methods. The best AUC in the dataset and the phylogenetic profiling methods (ASA or SEV) are shown in bold.

Phylogenetic profiling	Dataset	Ancestral state reconstruction	PRACU
ASA	Pseudomonadasles	MPPA	**0.316**
		DOWNPASS	0.257
		ACTRAN	0.296
		DELTRAN	0.290
	Mycobacteriales	MPPA	**0.192**
		DOWNPASS	0.098
		ACTRAN	0.158
		DELTRAN	0.146
	Archaea	MPPA	**0.134**
		DOWNPASS	0.132
		ACTRAN	0.133
		DELTRAN	0.118
SEV	Pseudomonadasles	DOWNPASS	0.371
		ACTRAN	**0.375**
		DELTRAN	0.370
	Mycobacteriales	DOWNPASS	0.251
		ACTRAN	**0.257**
		DELTRAN	0.251
	Archaea	DOWNPASS	0.187
		ACTRAN	**0.188**
		DELTRAN	0.185

Supplementary Table S2. The number of positive pairs detected by each phylogenetic profiling method in the Pseudomonadales dataset with varying true positive rates. The number of positive pairs detected by either weighted and transition methods are also shown. The ratios of the number of pairs detected by each method are shown in parentheses.

**TPR**	**0.9**	**0.8**	**0.7**	**0.6**	**0.5**
naive	1 (0.1%)	99 (6.4%)	888 (40.9%)	1073 (42.2%)	1186 (43.6%)
RLE	358 (49.4%)	1039 (66.8%)	1348 (62.0%)	1588 (62.4%)	1670 (61.4%)
CWA	265 (36.6%)	1040 (66.9%)	1630 (75.0%)	1844 (72.5%)	1983 (73.0%)
ASA	465 (64.2%)	1111 (71.4%)	1497 (68.9%)	1847 (72.7%)	2026 (74.5%)
weighted methods	478 (66.0%)	1193 (76.7%)	1706 (78.5%)	1980 (77.9%)	2128 (78.3%)
cotransitions	**619 (85.5%)**	1294 (83.2%)	1966 (90.5%)	2378 (93.5%)	**2567 (94.4%)**
SEV	609 (84.1%)	**1448 (93.1%)**	**2073 (95.4%)**	**2379 (93.6%)**	2515 (92.5%)
transition methods	666 (92.0%)	1457 (93.7%)	2111 (97.1%)	2432 (95.7%)	2595 (95.5%)
Total	724	1555	2173	2542	2718

Supplementary Table S3. The number of positive pairs detected by each phylogenetic profiling method in the Mycobacteriales dataset with varying true positive rates. The details are the same as Supplementary Table S3

TPR	0.9	0.8	0.7	0.6	0.5
naive	85 (42.7%)	221 (34.1%)	320 (28.9%)	439 (35.6%)	509 (39.5%)
RLE	127 (63.8%)	251 (38.7%)	303 (27.3%)	334 (27.1%)	342 (26.5%)
CWA	123 (61.8%)	298 (45.9%)	365 (32.9%)	435 (35.3%)	475 (36.8%)
ASA	**174 (87.4%)**	316 (48.7%)	607 (54.8%)	676 (58.9%)	802 (62.1%)
weighted methods	195 (98.0%)	421 (63.5%)	671 (60.1%)	784 (63.6%)	856 (66.4%)
cotransitions	107 (53.8%)	514 (79.2%)	988 (89.2%)	1124 (91.2%)	1192 (92.4%)
SEV	112 (56.3%)	**533 (82.1%)**	**1034 (93.3%)**	**1147 (92.4%)**	**1202 (93.1%)**
transition methods	117 (58.8%)	579 (89.2%)	1056 (95.3%)	1184 (96.0%)	1237 (95.9%)
Total	199	649	1108	1233	1290

Supplementary Table S4. The number of positive pairs detected by each phylogenetic profiling method in the Archaea dataset with varying true positive rates. The details are the same as Supplementary Table S3

**TPR**	**0.9**	**0.8**	**0.7**	**0.6**	**0.5**
naive	23 (3.7%)	272 (32.5%)	365 (38.1%)	418 (37.8%)	485 (40.3%)
RLE	326 (53.6%)	372 (44.4%)	396 (41.3%)	426 (38.7%)	447 (37.1%)
CWA	363 (59.7%)	469 (56.0%)	527 (55.0%)	590 (53.6%)	667 (55.4%)
ASA	287 (47.2%)	501 (59.9%)	605 (63.1%)	691 (62.8%)	748 (62.1%)
weighted methods	456 (75.0%)	633 (75.6%)	724 (75.5%)	807 (73.3%)	865 (71.8%)
cotransitions	515 (84.7%)	708 (84.6%)	805 (83.9%)	889 (80.7%)	1019 (84.6%)
SEV	**536 (88.2%)**	**734 (87.7%)**	**838 (87.4%)**	**985 (89.5%)**	**1065 (88.5%)**
transition methods	556 (91.4%)	763 (91.2%)	867 (90.4%)	1005 (91.2%)	1095 (90.9%)
Total	199	649	1108	1233	1290

Supplementary Table S5. The number of positive pairs only identified by each phylogenetic profiling method in the Pseudomonadales dataset with varying true positive rates. The number of positive pairs detected by either weighted methods but not the transition methods, and vice versa, are also shown. The ratios of the number of pairs detected by each method are shown in parentheses.

TPR	0.9	0.8	0.7	0.6	0.5
naive	0 (0%)	3 (0.2%)	5 (0.2%)	7 (0.3%)	5 (0.2%)
RLE	5 (0.7%)	7 (0.5%)	7 (0.3%)	11 (0.4%)	7 (0.3%)
CWA	0 (0%)	5 (0.3%)	11 (0.5%)	21 (0.8%)	29 (1.0%)
ASA	22 (3.0%)	24 (1.5%)	4 (0.2%)	24 (0.9%)	24 (0.9%)
weighted methods	58 (8.0%)	98 (6.3%)	62 (2.9%)	110 (4.3%)	123 (4.5%)
cotransitions	52 (7.2%)	5 (0.3%)	19 (0.9%)	30 (1.2%)	49 (1.8%)
SEV	42 (5.8%)	150 (9.6%)	144 (6.6%)	52 (2.0%)	26 (1.0%)
transition methods	246 (34%)	362 (23.2%)	467 (21.4%)	562 (22.1%)	590 (21.7%)

Supplementary Table S6. The number of positive pairs only identified by each phylogenetic profiling method in the Mycobacteriales dataset with varying true positive rates. The details are the same as Supplementary Table S3.

**TPR**	**0.9**	**0.8**	**0.7**	**0.6**	**0.5**
naive	16 (8.0%)	23 (3.5%)	10 (0.9%)	6 (0.5%)	5 (0.4%)
RLE	3 (1.5%)	9 (1.4%)	9 (0.8%)	9 (0.7%)	8 (0.6%)
CWA	1 (0.5%)	8 (1.2%)	5 (0.5%)	5 (0.4%)	10 (0.8%)
ASA	18 (9.0%)	5 (0.8%)	12 (1.0%)	7 (0.6%)	12 (1.0%)
weighted methods	82 (41.2%)	70 (1.1%)	52 (4.7%)	49 (4.0%)	53 (4.1%)
cotransitions	0 (0%)	32 (5.0%)	11 (1.0%)	24 (2.0%)	25 (1.9%)
SEV	3 (1.5%)	59 (9.0%)	61 (5.5%)	58 (4.7%)	43 (3.3%)
transition methods	4 (2.0%)	237 (36.5%)	437 (39.4%)	449 (36.4%)	434 (33.6%)

Supplementary Table S7. The number of positive pairs only identified by each phylogenetic profiling method in the Archaea dataset with varying true positive rates. The details are the same as Supplementary Table S3.

TPR	0.9	0.8	0.7	0.6	0.5
naive	5 (0.8%)	13 (1.5%)	17 (1.8%)	12 (1.1%)	17 (1.4%)
RLE	12 (2.0%)	13 (1.5%)	13 (1.4%)	12 (1.1%)	10 (0.8%)
CWA	16 (2.6%)	19 (2.3%)	10 (1.0%)	16 (1.5%)	16 (1.3%)
ASA	3 (0.5%)	15 (1.8%)	24 (2.5%)	31 (2.8%)	26 (2.1%)
weighted methods	52 (8.6%)	74 (8.8%)	92 (9.6%)	96 (4.0%)	109 (9.1%)
cotransitions	8 (1.3%)	14 (1.7%)	15 (1.6%)	6 (0.5%)	16 (1.3%)
SEV	34 (5.6%)	48 (5.7%)	54 (5.6%)	103 (9.4%)	74 (6.1%)
transition methods	152 (25.0%)	204 (24.4%)	235 (24.5%)	294 (36.4%)	339 (28.2%)
