# Supplementary material for: CORGIAS: identifying correlated gene pairs by considering evolutionary history in a large-scale prokaryotic genome dataset
Source: NAR Genom Bioinform. 2025 Dec 12;7(4):lqaf182. doi: 10.1093/nargab/lqaf182 (PMC12699329; doi:10.1093/nargab/lqaf182)
Supplement: lqaf182_Supplemental_Files [file lqaf182_supplemental_files.zip › TableS11.pdf]

Supplementary Table S11. Runtime regression of the phylogenetic profiling methods used in this study. Runtimes (t) were regressed on the following: $\log(t) = \alpha_1 \log(\text{number of species}) + \alpha_2 \log(\text{number of genes}) + \beta$. The predicted runtimes of the latest GTDB (Release 220, 107,000 species and 6,000 OGs) and OrthoDB (v12, 28,000 species, and 61,000 OGs) are also shown. The numbers of OGs were obtained from previous studies (Tremblay *et al.* 2021, Dembech *et al.* 2023).

	α_1	α_2	β	R^2	OrthoDB	GTDB
naïve	1.52	2.23	-25.6	0.990	31 d	54 h
naïve w/ GPU	0.219	1.52	-12.2	0.979	5.8 m	10 m
RLE	0.205	2.02	-12.0	0.999	19 h	51 h
CWA	0.905	2.00	-15.4	0.997	63 d	22 d
ASA	1.09	1.85	-13.2	0.999	2.9 y	195 d
cotransitions	0.847	1.79	-17.6	0.982	10.7 h	3.8 h
cotransitions w/ GPU	0.336	1.27	-10.7	0.951	8.3 m	8.4 m
SEV	0.884	1.851	-17.7	0.998	27 h	9.0 h
SEV w/ GPU	0.521	1.61	-14.5	0.954	48 m	38 m
G/L Distance	1.091	2.01	-17.2	0.992	52 d	11d
